# Use of a Florida Gulf Coast Barrier Island by Spring Trans-Gulf Migrants and the Projected Effects of Sea Level Rise on Habitat Availability

**DOI:** 10.1371/journal.pone.0148975

**Published:** 2016-03-02

**Authors:** Lori A. Lester, Mariamar Gutierrez Ramirez, Alan H. Kneidel, Christopher M. Heckscher

**Affiliations:** NOAA Environmental Cooperative Science Center, Department of Agriculture and Natural Resources, Delaware State University, Dover, Delaware, United States of America; Cornell University, UNITED STATES

## Abstract

Barrier islands on the north coast of the Gulf of Mexico are an internationally important coastal resource. Each spring hundreds of thousands of Nearctic-Neotropical songbirds crossing the Gulf of Mexico during spring migration use these islands because they provide the first landfall for individuals following a trans-Gulf migratory route. The effects of climate change, particularly sea level rise, may negatively impact habitat availability for migrants on barrier islands. Our objectives were (1) to confirm the use of St. George Island, Florida by trans-Gulf migrants and (2) to determine whether forested stopover habitat will be available for migrants on St. George Island following sea level rise. We used avian transect data, geographic information systems, remote sensing, and simulation modelling to investigate the potential effects of three different sea level rise scenarios (0.28 m, 0.82 m, and 2 m) on habitat availability for trans-Gulf migrants. We found considerable use of the island by spring trans-Gulf migrants. Migrants were most abundant in areas with low elevation, high canopy height, and high coverage of forests and scrub/shrub. A substantial percentage of forest (44%) will be lost by 2100 assuming moderate sea level rise (0.82 m). Thus, as sea level rise progresses, less forests will be available for migrants during stopover. Many migratory bird species’ populations are declining, and degradation of barrier island stopover habitat may further increase the cost of migration for many individuals. To preserve this coastal resource, conservation and wise management of migratory stopover areas, especially near ecological barriers like the Gulf of Mexico, will be essential as sea levels rise.

## Introduction

Climate change is an important conservation concern for coastal ecosystems. Sea level rise (SLR) is expected to accelerate due to global warming, particularly in low elevation coastal areas such as barrier islands [1,2]. As global temperatures have increased, sea levels have risen approximately 15 cm during the 20th century [3]. Sea levels will likely continue rising due to thermal expansion of ocean waters and melting of glaciers and ice sheets [4]. The most recent Intergovernmental Panel on Climate Change (IPCC) report predicts that sea level will rise 26 to 82 cm by 2100 [5], and that this amount of rise will likely lead to inundation of coastal ecosystems and to changes in habitat availability for animal species [6–8].

Each spring, many Nearctic-Neotropical passerine migrants undertake a flight across the Caribbean Sea and the Gulf of Mexico from wintering grounds in Central or South America to breeding grounds in North America. A large percentage of North America’s breeding songbirds migrate across the Gulf of Mexico [9–11] and some also cross the Caribbean Sea with nonstop flights often exceeding 1000 km [12]. Barrier islands along the northern coast of the Gulf of Mexico often provide the first potential stopover habitat for birds following a trans-Gulf migration [9,13]. Therefore, Gulf Coast barrier islands are of international conservation importance as refueling sites for Nearctic-Neotropical migrants and can provide birds with an essential place to rest while avoiding predators including raptors that may also concentrate in coastal areas [14]. This international coastal resource, akin to better known shorebird migratory concentration sites [15], is often overlooked by environmental managers because the small songbirds rest and forage by the thousands, often out of sight in dense vegetation, and there can be substantial spatial and temporal variation in numbers among years [16,17].

Annual survivorship of migrants are strongly influenced by high rates of mortality during migratory periods, and stopover sites are essential to the maintenance of Nearctic-Neotropical migrant populations [18]. Complicating matters, migrants visiting stopover sites oftentimes do not have adequate energy (e.g., fat stores) to search for the best available stopover site and are forced to use sub-optimal sites [19–21]. For example, during stopover, lean Summer Tanagers (*Piranga rubra*) are more active than fatter birds because they explore a wider variety of habitats while searching for food resources [22]. Therefore, studying and preserving coastal migratory stopover habitat will be essential components of understanding the full annual cycle of migratory passerines, and these studies are desperately needed to identify limiting factors affecting North American breeding populations [23].

Our goal was to confirm the use of a Gulf coast barrier island (St. George Island, Florida, USA) by trans-Gulf migrant passerines and to investigate the potential effects of future SLR on their stopover habitat. While studies of migrants along the west and central Gulf coast have been underway for many years [9], our study may be the first to empirically examine the use of barrier islands by trans-Gulf migrants on the eastern side of the Gulf of Mexico. Traditionally, this region has been considered less important as an internationally significant stopover resource [24]. To achieve our goal, we created habitat land cover maps from satellite images to estimate habitat type coverage on the island. We also conducted avian surveys along transects to determine which habitat types migrants utilized during our study period. Finally, we modeled the potential impacts of SLR on habitat currently available to migrants.

## Materials and Methods

### Ethics Statement

A Special Use Permit (FF04RFSM00) was provided by the US Fish and Wildlife Service. No migrants were collected, sampled, or handled during this study.

### Study Site

The Apalachicola Bay barrier island chain contains four islands: Dog Island, St. George Island, Little St. George Island, and St. Vincent Island. St. George Island is located in this northwest Florida barrier island chain in Franklin County about 112 km southwest of Tallahassee (29.671479°, -84.838581°). The island is 45 km long and 2 km at the widest point. Although inhabited by approximately 3,300 people, the eastern 14 km of the island is protected from development as the Dr. Julian G. Bruce St. George Island State Park. The island is approximately 8 km from the mainland and is the southern boundary of the Apalachicola Bay.

**Fig 1:**
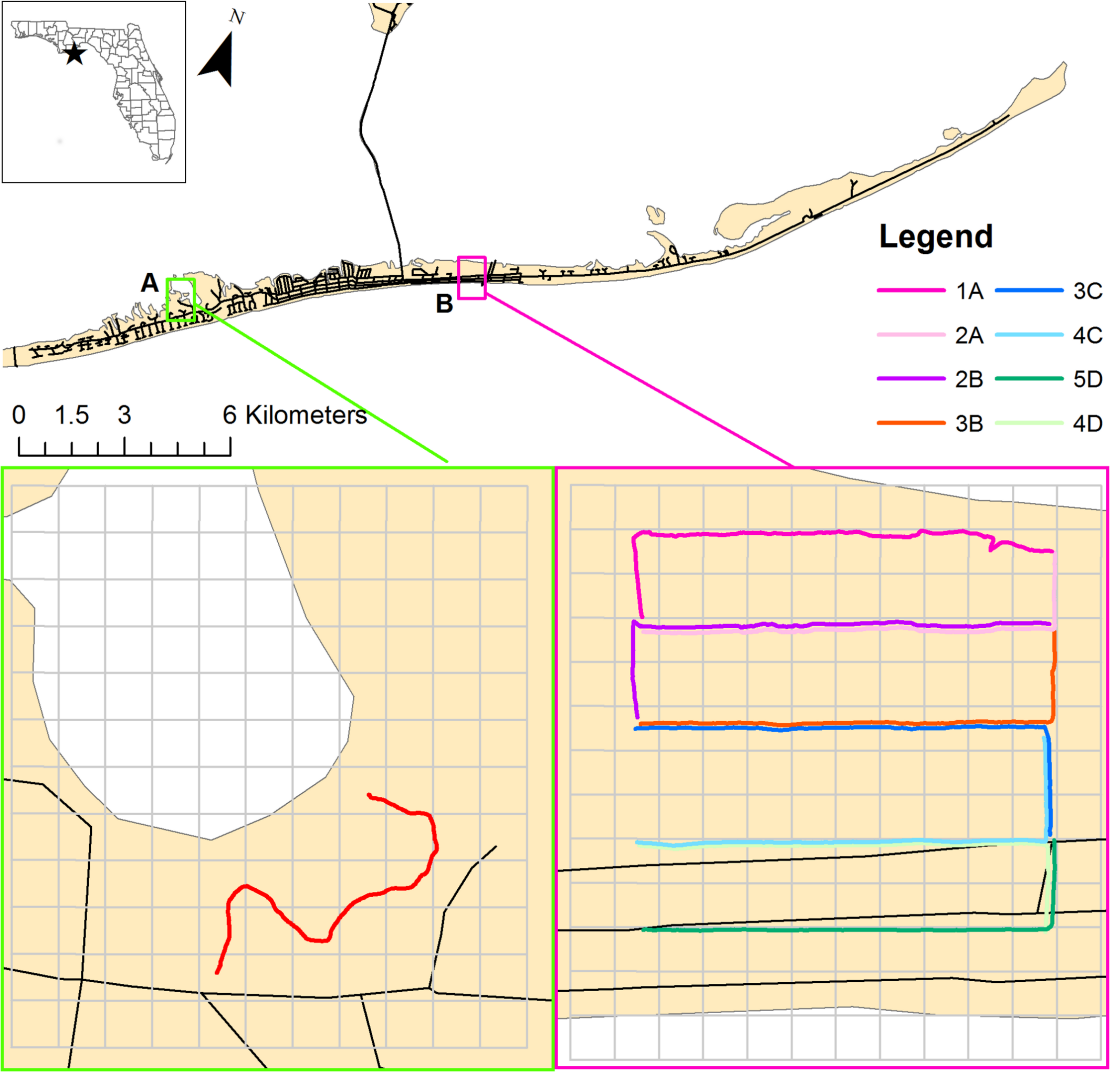
Transects on St. George Island, Florida, USA. Transects were conducted at two study sites: (A) Nick’s Hole and (B) Unit 4. Observers detected migrants by sight and/or sound while walking nine transects: one in Nick’s Hole and eight in Unit 4. Avian abundance was calculated as number of migrants observed divided by transect length in each cell.

We collected data during two field seasons from 19 April to 10 May, 2013 and 13 April to 9 May 2014. We focused on two study sites: Nick’s Hole and Unit 4 (Fig 1). Both sites are in the Apalachicola National Estuarine Research Reserve (ANERR) and designated as Coastal and Aquatic Managed Areas (CAMA), and thus managed by the Florida Department of Environmental Protection (DEP).

### Transects

In order to determine habitat use of migrants, we conducted nine transects through different habitat types. Each transect was 500 m in length, with eight located in Unit 4 and one in Nick’s Hole (Fig 1). The Nick’s Hole transect was selected because it contained large patches of palmetto plants (*Sabal palmetto*), and these patches were not available in Unit 4. One observer per day conducted transects in the early evenings (1630 to 1830), and walked each transect seven times in 2013 and four times in 2014 (99 transects total). The order in which transects were conducted was randomized with a block design, so that no two adjacent transects were walked in the same day. This ensured that transects were at least 100 m apart to avoid oversampling. Along the Gulf coast, there is high variability in numbers of migrants present on any given day during migration. It was not feasible to conduct each transect each day during migration, thus this variability is a limitation of our study. We chose to survey birds in the evening because most trans-Gulf migrants arrive in late morning or early afternoon and by evening should be settled in preferred habitat and actively foraging in preparation for an early evening departure. The observer walked at a slow, steady pace while recording each migrant detected by sight and/or sound. Observers recorded species, distance from transect, height of first detection, and the general type of vegetation cover in which the bird was observed (S1 Data). In addition, observers referred to a 50 m x 50 m grid that was overlaid on a high-resolution aerial photograph. Observers recorded the grid cell in which each migrant was located. To obtain an index of avian relative abundance for each grid cell along transects, we calculated the total mean number of migrants per count observed in each cell. Transects were not straight lines, and thus some cells contained more or less than 50 m of transect. Therefore, we divided mean migrants per count by transect distance per cell to control for variation in distance walked by the observers in each cell. Although observer ability to detect songbirds undoubtedly decreased as distance of the birds from transect increased, we assumed our ability to detect birds remained constant for each cell providing a reliable index to avian abundance.

### Habitat Maps

We acquired WorldView-2 (WV2) high-resolution satellite imagery of St. George Island, Florida that was taken on 6 May 2013 (Digital Globe, Longmont, Colorado, USA). The WV2 imagery consists of one panchromatic band with 0.5 m resolution and eight multispectral bands with 2 m resolution. We performed an atmospheric correction with the ENVI module (ENVI 4.8, Exelis Visual Information Solutions, Boulder, Colorado, USA) QUick Atmospheric Correction (QUAC) in ArcMap 10 to retrieve spectral reflectance from the multispectral image.

We viewed and classified the WV2 image using the processing software Erdas Imagine 2010. The study sites were classified into eight landcover categories (mixed forest, sand, marsh, palmetto, grass, scrub/shrub, water, and urban) based on spectral signatures. We performed a supervised classification to organize data into the landcover categories by defining three Areas of Interest (AOI) polygons for each category at both study sites. The AOI polygons were recorded by observer GPS survey using Trimble Nomad 900 L units (Trimble Navigation Limited, Sunnyvale, California, USA). The supervised classification used the Gaussian Maximum Likelihood (GML) algorithm to assign each pixel in the WV2 image to the most likely landcover category based on spectral pattern. We then performed a neighborhood classification smoothing to analyze class values based on surrounding pixel class values with a 3 x 3 cell moving window.

For accuracy assessment of classification maps, we generated and assigned to classes 256 random points for each study site. We determined the UTM coordinates of each point, navigated to each point, and recorded actual habitat type. An error matrix for each map was then generated by comparing classified classes to actual classes on each map, and overall accuracy was measured. From the classification maps, we calculated area of habitat cover of each type in all grid cells in ArcMap 10. The percent habitat cover for each type and cell was then calculated (S1 Data).

### Canopy and Understory Height

We measured canopy and understory heights in both study sites using the clinometer method [25]. While walking each transect, we estimated canopy and understory height every 50 m by measuring the angle between an observer’s eye and the top of the vegetation and the distance from the observer to the vegetation with a rangefinder (Bushnell Scout DX 1000 Arc, Overland Park, Kansas, USA). Canopy and understory heights (*H*) were calculated as:

H=b*tan(θ)+e

where *b* was distance on the ground from observer to tree, *θ* was angle from observer’s eye to the top of the tree, and *e* was distance from the ground to the observer’s eye (S1 Data).

### Elevation

Real-time kinematic (RTK) GPS surveys were conducted using a Trimble R8 Global Navigational Satellite System (GNSS; Trimble Navigation Limited, Sunnyvale, California, USA). Elevation was measured at 3 m to 30 m intervals in Nick’s Hole and 2 m to 10 m intervals at Unit 4 along the nine transects. Measurement interval was not consistent because of signal interference from tree canopy.

### Sea Level Rise Model

We used the Sea Level Affecting Marshes Model (SLAMM 6, Warren Pinnacle Consulting, Inc., Waitsfield, VT) to predict the effect of SLR on habitat availability for migrants on St. George Island, Florida. This model simulates dominant processes involved in wetland conversion and shoreline modification during SLR. The six dominant processes considered were inundation, erosion, overwash, saturation, accretion, and salinity. Our raster inputs (10 m resolution) included the USGS National Elevation Dataset (NED; http://ned.usgs.gov), slope derived from NED, and Cooperative Land Cover (CLC; Version 2.3; www.fnai.org). The CLC classes were converted to SLAMM categories in ArcMap 10.2 (S1 Table). We also included non-spatial, site specific input parameters including dates of aerial photos, direction offshore, historic trend in SLR, North American Vertical Datum (NAVD) Correction, tidal elevation, erosion rates, accretion rates, and beach sedimentation rate (Table 1).

**Table 1:** Environmental Parameters for Sea Level Affecting Marshes Model.

Parameter	Values	Reference
**Landcover Photo Date**	2009	Cooperative Land Cover
**DEM Date**	2009	USGS National Elevation Dataset
**Direction Offshore**	South	
**Historic Trend**	1.38 mm/yr	http://tidesandcurrents.noaa.gov Station 8728486
**NAVD Correction**	0.006 m	http://tidesandcurrents.noaa.gov
**Great Diurnal Tide Range**	0.67 m	http://tidesandcurrents.noaa.gov
**Salt Elevation**	0.4 m	http://tidesandcurrents.noaa.gov
**Marsh Erosion Rate**	1 m/yr	(Schneider and Kruse 2005)
**Swamp Erosion Rate**	1 m/yr	(Schneider and Kruse 2005)
**Tidal Flat Erosion**	1 m/yr	(Schneider and Kruse 2005)
**Salt Marsh Accretion Rate**	2.25 mm/yr	(Clough 2006)
**Brackish Marsh Accretion Rate**	3.75 mm/yr	(Clough 2006)
**Tidal Freshwater Marsh Accretion Rate**	4 mm/yr	(Clough 2006)
**Frequency of Overwash**	25 years	

In addition to spatial inputs, these non-spatial, site-specific input parameters were included in the sea level rise models for St. George Island, Florida.

We based two of our model simulations on the Representative Concentration Pathway (RCP) scenarios [5]. In particular, we used the minimum increase in SLR from RCP 2.6 (0.26 m) and the maximum increase from RCP 8.5 (0.82 m). The RCP 2.6 is representative of a major reduction in greenhouse gas emissions and the RCP 8.5 is characterized by increasing greenhouse gas emissions over time. We also completed a simulation with 2 m of SLR by 2100 because the RCP scenarios likely underestimate SLR because they do not take into account calving which is when large pieces of ice break off at the terminus of a glacier causing faster melting than expected [26–28].

### Statistical Analysis

A generalized linear mixed effects model (GLMM) was used to determine whether migrant abundance was biased among habitat characteristics because migrant abundance was not normally distributed. The random effects included grid cell, transect, and site as repeated variables, whereas the fixed effects included year, elevation, canopy and understory height, and percent habitat coverage per cell (forest, sand, marsh, palmetto, grass, scrub, water, and urban). We used restricted maximum likelihood (REML) to model changes in migrant abundance based on habitat characteristics with the package lme4 in program R 3.2.2 [29]. We obtained *P*-values by likelihood ratio tests of the full model against the model without the effects of interest. Furthermore, we ran a Student’s *t*-test to determine if elevation differed between sites and a MANOVA to test for differences in canopy and understory heights between sites. Statistical significance was accepted when *P* ≤ 0.05.

## Results

### Habitat Use by Nearctic-Neotropical Migrants

**Fig 2:**
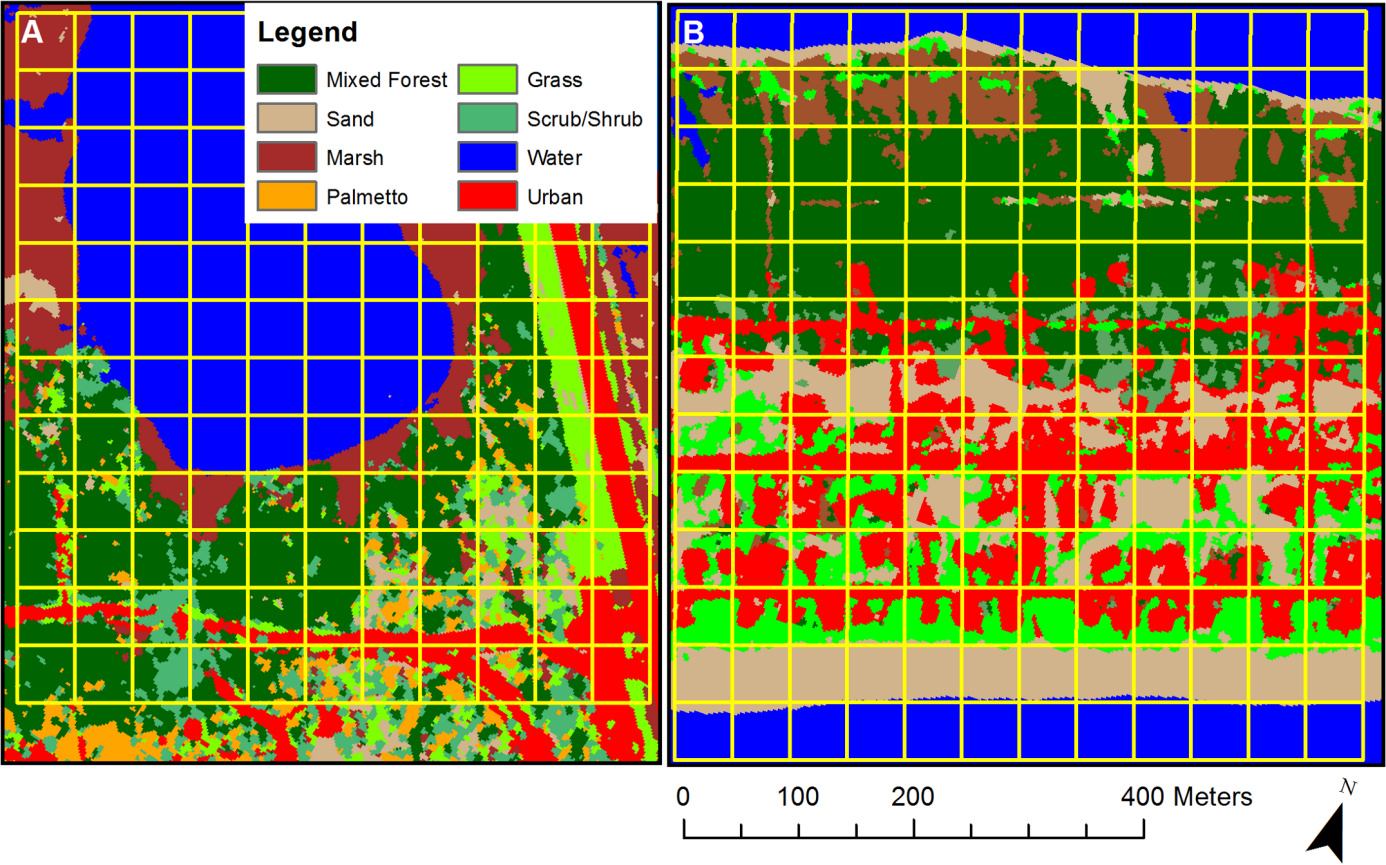
Habitat Maps. Habitat maps of sites were created using the supervised classification method on WorldView-2 satellite imagery. Percent habitat type coverage was calculated for each cell and compared to avian abundance.

We created habitat maps of our two study sites from the WorldView-2 satellite imagery (Fig 2), and accuracy was 95.3% for Nick’s Hole and 91.4% for Unit 4. While conducting transects, we detected 711 individuals of 57 different transient species (See Table 2 for common species). Migrant abundance was biased to certain habitat types. The best fit model for predicting migrant abundance accounted for elevation, canopy height, percent forest cover, and percent scrub cover (GLMM; Tables 3 & 4). Migrants were most common in areas with low elevation, high canopy height, and high coverage of forests or scrub/shrub habitats (Likelihood Ratio Test; *P* < 0.001).

**Table 2:** Most Common Nearctic-Neotropical Migrant Species Detected Visually or Audibly by Observers during Transects.

*n*	Common Name	Scientific Name
162	Gray Catbird	*Dumetella carolinensis*
69	Indigo Bunting	*Passerina cyanea*
38	Red-eyed Vireo	*Vireo olivaceus*
37	Common Yellowthroat	*Geothlypis trichas*
36	Rose-breasted Grosbeak	*Pheucticus ludovicianus*
32	Great Crested Flycatcher	*Myiarchus crinitus*
30	Summer Tananger	*Piranga rubra*
26	Hooded Warbler	*Setophaga citrina*
25	Black-throated Green Warbler	*Setophaga virens*
24	Yellow Warbler	*Setophaga petechia*

**Table 3:** Best Fit Model Selection.

Model	Parameters	AIC	BIC	Log Likelihood
Full	Year + Elevation + Canopy + Forest + Scrub + Understory + Sand + Marsh + Palmetto + Grass + Water + Urban	-209.2	-156.3	121.6
Reduced	Year + Understory + Sand + Marsh + Palmetto + Grass + Water + Urban	-208.7	-168.3	117.4
Best Fit	Elevation + Canopy + Forest + Scrub	-235.3	-148.2	145.7

**Table 4:** Results from Best Fit Generalized Linear Mixed Effects Model (GLMM).

Model Parameter(s)	Estimate	SE	*df*	*F*-value
Elevation	-0.038	0.03	1,28	14.66
Canopy	0.003	0.002	1,28	0.01
Forest	0.003	0.0006	1,28	15.34
Scrub	0.006	0.002	1,28	7.58

The mixed forest on St. George Island is comprised of a canopy of slash pine (*Pinus elliotii*) and an evergreen understory of live oak (*Quercus virginiana*), sand live oak (*Quercus geminata*), yaupon (*Ilex vomitoria*), wax myrtle (*Myrica cerifera*), and other less abundant plants. Mean canopy height was significantly higher at Nick’s Hole than Unit 4 (Table 5; MANOVA, *P* = 0.002); however, mean understory height was similar between sites (MANOVA, *P* = 0.4). The elevation was significantly higher at Unit 4 (1.6 m) than Nick’s Hole (1.2 m; Table 5; Student’s *t* test, *P* < 0.001). On St. George Island, many higher elevation areas have been developed and thus forested areas are more common in lower elevation areas.

**Table 5:** Comparison of Habitat Characteristics among Sites.

Variable	Summary Statistic	Nick’s Hole	Unit 4	Test Statistic	*P*-value
Canopy	Mean (m)	17.4 ± 1.6 SE	12.1 ± 0.6 SE	*F*_*(1*,*57)*_ = 10.8	0.002
	Min (m)	1.6	0.0		
	Max (m)	36.2	20.7		
Understory	Mean (m)	2.3 ± 0.3 SE	2.6 ± 0.3 SE	*F*_*(1*,*57)*_ = 0.7	0.4
	Min (m)	0.0	0.0		
	Max (m)	6.6	6.0		
Elevation	Mean (m)	1.20 ± 0.03 SE	1.58 ± 0.03 SE	*T* = 8.9	< 0.001
	Min (m)	0.40	0.18		
	Max (m)	1.84	2.78		

### SLAMM

**Fig 3:**
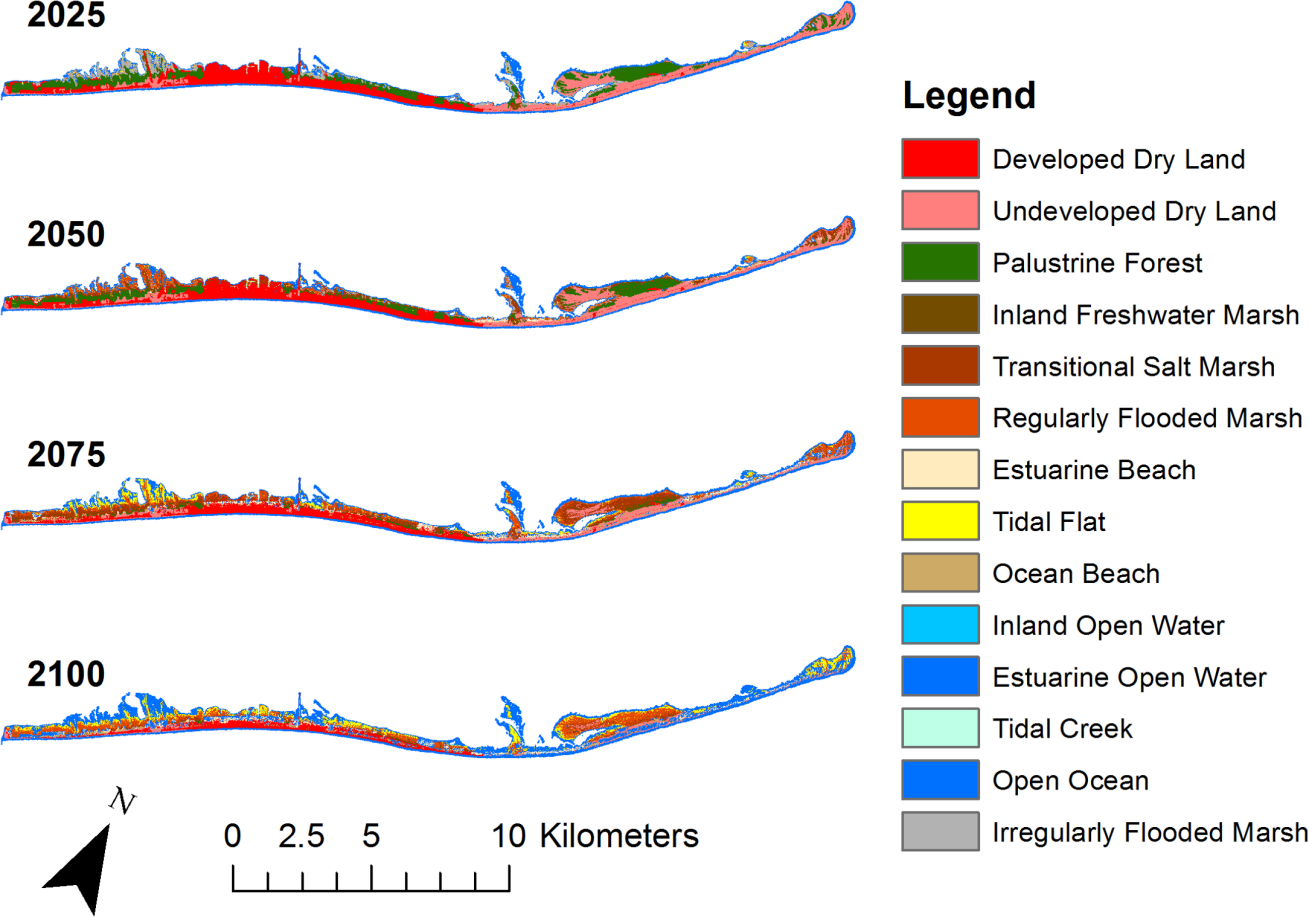
Results of Sea Level Affecting Marshes Model (SLAMM 6). The SLAMM simulation modeling results for 2 m sea level rise scenario predict that 98% of palustrine forested area will be lost between 2009 and 2100.

Simulation modeling predicts that SLR will lead to substantial declines in palustrine forest cover on St. George Island, Florida (Table 6; Fig 3). For this island, the RCP 8.5 model (0.82 m SLR) predicted a large decline in area of palustrine forest (45%) and inland freshwater marsh (46%) by 2100. Corresponding increases will occur in marsh cover (transitional salt marsh, 1205%; regularly flooded marsh, 514%). A 0.26 m increase in sea level, predicted by the minimum RCP 2.6 scenario, would lead to lesser reduction in palustrine forest cover on St. George Island with only 4% lost by 2100. However, the prediction for a 2 m SLR would be 98% palustrine forest lost over the same time period. In general, forested areas are predicted to transition to marsh, and then erode to tidal flat.

**Table 6:** Sea Level Affecting Marshes Model (SLAMM 6.0).

	Area (km^2^)	% Change (0.26 m)	% Change (0.82 m)	% Change (2 m)
Palustrine Forest	4.83	-4	-45	-98
Developed Dry Land	5.34	-2	-14	-58
Undeveloped Dry Land	4.64	-2	-19	-77
Inland Fresh Marsh	0.07	-10	-46	-99
Transitional Salt Marsh	0.12	205	1205	576
Regularly Flooded Marsh	0.31	86	514	1001
Ocean Beach	1.91	-2	-95	-69
Estuarine Open Water	2.83	1	15	111
Tidal Creek	0.04	0	0	0
Open Ocean	0.39	16	577	1117
Irregularly Flooded Marsh	1.97	-14	-74	-100

Baseline areas (km^2^) are presented for each habitat class in 2009. Percent change in habitat cover between 2009 and 2100 was then calculated for three different sea level rise scenarios (0.26 m, 0.82 m, and 2 m). The SLAMM predicts that substantial declines in palustrine forest cover will occur on St. George Island, Florida by 2100.

## Discussion

Our results are the first to empirically confirm that St. George Island, a large barrier island formation in the Apalachicola Bay, is used consistently by a high diversity and abundance of trans-Gulf migrants during spring migration. St. George Island is comparable in regards to species diversity to western and central Gulf coast migratory stopover sites in Texas, Louisiana, Mississippi, and Alabama. For example, species diversity was between 49 and 59 species per year captured and banded on East Ship Island, MS [20] and 46 species detected using point counts on Horn Island, MS [9].

We determined that Neotropical migrants on St. George Island were associated with forest and scrub/shrub habitats, and these results are similar to those of other studies from barrier islands on the northern coast of the Gulf of Mexico. For example, migrants on Horn Island, MS were found to select scrub/shrub, pine forest, and relic dune habitats over primary dune or marsh/meadow habitats [9]. The amount of forest cover was not only positively correlated with migrant density at those sites, but also with food resources such as arthropods [30,31]. Migrants may use forest cover as a cue to foraging habitat following a trans-Gulf flight.

We found that migrant abundance was greater in low elevation areas that consisted of mixed forest. Mixed forest is presumably beneficial at stopover sites because it provides protection from predators and more abundant food resources than sparse or monotypic understory [9]. Low elevation areas are often covered in forest because higher elevation areas on St. George Island have been affected by residential development. Our models suggest that these low elevation, high canopy height, forested and scrub/shrub areas, usually consisting of mixed forest, are particularly likely to be negatively impacted by future SLR.

Our study is the first to assess the threat of SLR on barrier islands for Nearctic-Neotropical migratory bird stopover. Future SLR due to climate change will negatively affect barrier island stopover habitat availability for migrants using the Apalachicola Bay barrier islands. By 2100, many barrier islands along the coast of the Gulf of Mexico will experience major transitions in habitat availability including the conversion of palustrine forest to marshes on St. George Island. Migrant abundance on St. George Island was low in marsh habitats, and similar results have been found on Horn Island, MS [9]. Therefore, adjacent inland forested habitats must be protected from development to increase the probability that forested stopover habitat will be available for migrants despite SLR.

As with all models, the SLAMM results are subject to limitations from data inputs, incomplete knowledge about factors, and oversimplification of the system. The NED elevation data available for St. George Island was moderate resolution (10 m), and thus cell size of all spatial inputs were at this resolution. Moreover, hurricanes will have a profound impact on stopover habitat availability for migrants on St. George Island, FL, and the timing and size of future storms is uncertain. In 2004 and 2005, Hurricanes Ivan and Dennis overwashed the eastern portion of the island, inundating some forested areas with sediment and saltwater [32,33]. Although our model accounted for an overwash every 25 years, hurricane activity has increased recently due to rising North Atlantic sea surface temperatures and decreases in vertical wind shear [34]. This swell in number of hurricanes could cause more frequent overwash and should be considered when managing barrier islands. Despite these limitations, our analysis provides important insights into how SLR may affect stopover habitat availability for migrants.

Although some conservation actions have been taken on avian breeding and wintering grounds, limited focus has been placed on stopover sites [35]. Migration is the most unpredictable period of the avian annual cycle [36,37]. Mortality rates of songbird migrants are up to 15 times higher during migration compared to stationary periods of breeding and wintering [15,36,38]. If conservation measures are not taken to address losses of stopover habitat, efforts on breeding and wintering areas may be compromised. The additional threat of declining availability of mixed forests due to SLR for migrants during stopover could further exacerbate the problem. Degradation of barrier island habitat may increase the cost of migration because migrants may be forced to continue past the island to the mainland. From St. George Island, migrants would need to continue at least 8 km to reach the coastline which may be a significant distance for the individuals that have completely exhausted their pectoral muscles (S1 Photograph). Once on the mainland, ideal habitat (mature and dense mixed forest) may not be available for several more kilometers inland, especially because SLR will presumably also affect the mainland. Migrants experience declined energetic condition from crossing the Gulf of Mexico. Therefore, most migrants select stopover sites within 18 km of the coastline during first stopover after crossing the Gulf and these areas will be strongly affected by SLR [30]. Perhaps most importantly, barrier islands may provide essential “fire escape” habitat [35] for spring migrants during periods of strong northerly winds in which continued movement inland may be very costly [39,40]. Thus it is essential to protect and wisely manage important stopover areas such as St. George Island, particularly those before and after significant ecological barriers such as the Gulf of Mexico.

## Supporting Information

S1 Data Nearctic-Neotropical Songbird Transects and Habitat Variables. During transects, observers collected data on songbirds detected. Habitat variables including percent habitat cover, elevation, and canopy and understory heights were also collected.
(XLSX)

S1 Photograph An Emaciated Yellow-billed Cuckoo (*Coccyzus americanus*) Captured on St. George Island, FL during Spring Migration. (JPG)

S1 Table Cooperative Land Cover (CLC) Classes Converted to Sea Level Affecting Marshes Model (SLAMM) Categories. (DOCX)
