# Supplementary figures and images for: Use of a Florida Gulf Coast Barrier Island by Spring Trans-Gulf Migrants and the Projected Effects of Sea Level Rise on Habitat Availability
Source: PLoS One. 2016 Mar 2;11(3):e0148975. doi: 10.1371/journal.pone.0148975 (PMC4775044; doi:10.1371/journal.pone.0148975)

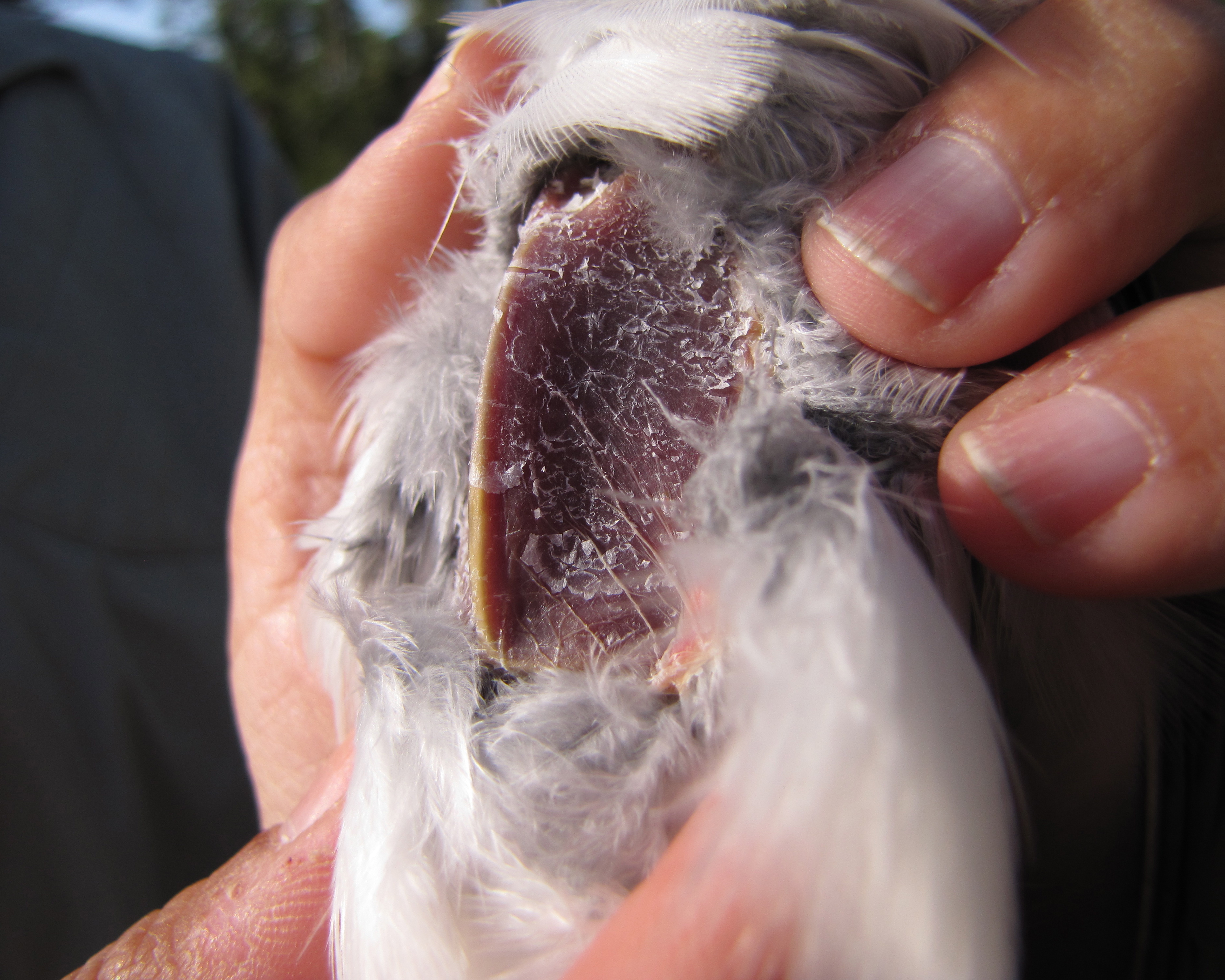

Supplement: S1 Photograph — (JPG) [file pone.0148975.s002.jpg]
