# Supplementary material for: Use of a Florida Gulf Coast Barrier Island by Spring Trans-Gulf Migrants and the Projected Effects of Sea Level Rise on Habitat Availability
Source: PLoS One. 2016 Mar 2;11(3):e0148975. doi: 10.1371/journal.pone.0148975 (PMC4775044; doi:10.1371/journal.pone.0148975)
Supplement: S1 Table — (DOCX) [file pone.0148975.s003.docx]

| **CLC Code** | **CLC Description** | **SLAMM Code** | **SLAMM Description** |
| --- | --- | --- | --- |
| 1821000 | Low Intensity Urban | 1 | Developed Dry Land |
| 1822000 | High Intensity Urban | 1 | Developed Dry Land |
| 1840000 | Transportation | 1 | Developed Dry Land |
| 1860000 | Utilities | 1 | Developed Dry Land |
| 1210000 | Scrub | 2 | Undeveloped Dry Land |
| 1610000 | Beach Dune/Coastal Grassland | 2 | Undeveloped Dry Land |
| 1830000 | Rural Lands | 2 | Undeveloped Dry Land |
| 1650000 | Maritime Hammock | 3 | Palustrine Forest |
| 2122000 | Coastal Interdunal Swale | 3 | Palustrine Forest |
| 2221000 | Wet Flatwoods | 3 | Palustrine Forest |
| 2100000 | Freshwater Non-Forested Wetlands | 5 | Inland Freshwater Marsh |
| 2110000 | Prairies and Bogs | 5 | Inland Freshwater Marsh |
| 2120000 | Freshwater Marshes | 5 | Inland Freshwater Marsh |
| 1670000 | Sand Beach | 12 | Ocean Beach |
| 3100000 | Natural Lakes and Ponds | 15 | Inland Open Water |
| 3200000 | Artificial Lakes and Ponds | 15 | Inland Open Water |
| 5000000 | Estuarine | 17 | Estuarine Water |
| 4100000 | Natural Rivers and Streams | 18 | Tidal Creek |
| 6000000 | Marine | 19 | Open Ocean |
| 5240000 | Saltwater Marsh | 20 | Irregularly Flooded Marsh |
